# Asthma and Its Relation to Nasal Diseases

**Published:** 1905-04-29

**Authors:** 


### ASTHMA AND ITS RELATION TO NASAL DISEASES.

Dr. W. Kent Hughes[1] considers that all congestive conditions of the nasal passages may be from time to time the cause of asthma, as may mucous polypi. He lays down three factors as operative in all asthmatics, namely:—1. Predisposition. 2. Deviation from perfect integrity of structure in some parts of the air passage, *i.e.* some undue excitability of mucous membrane. 3. A distant irritant. The most generally accepted theory of the attack itself attributes it to a reflex spasm of the muscles of the bronchioles, and Dr. Hughes says that experiment has repeatedly revealed the fact that stimulation of the mucosa of the nasal septum, especially in its upper and posterior part, is capable of producing bronchial spasm. He himself has found that on touching certain areas of the mucosa of the septum he has been able to induce either asthma, or sneezing, or cough, or a feeling of oppression.

The author quotes Francis to the effect that in all cases, even where no pathological condition of the nasal passages can be revealed, treatment of asthma should include the cauterisation of an area of the septum situated opposite the anterior third of the middle turbinate bone, and running downwards and forwards. This is reputed to have the effect of rendering stable a previously unstable respiratory centre. By this means Francis claims to have cured 75 per cent. of over 400 cases of asthma, many of them remaining free for six years and more. Dr. Hughes, while not unreservedly accepting the *rationale* of the system, is fully in accord with Francis as to its beneficial effects, and recommends the adoption of the practice in spite of its empiricism.

We venture to think that such treatment is not altogether so empirical as it would appear to be at first sight. When it is remembered that stimulation of the nasal mucous membrane can produce so complex a muscular reflex as is called for by an ordinary sneeze, it does not tax imagination very far to believe that less complex muscular contractions may depend upon slighter degrees of irritation in a subject whose reflex irritability is constitutionally enhanced. The better one's perspective of disease in general, the more obvious becomes the important part played by reflexes in the origin and perpetuation of spasmodic ailments. For instance, it is past all question that a common, if not the commonest, cause of laryngismus stridulus is post-nasal catarrh, generally in association with adenoids, and that the same condition is responsible for the bulk of "dry coughs" in childhood. Most observers agree that convulsive tics owe their inception as a rule to some reflex response to irritation, this response degenerating into a habit after the cessation of the stimulus; while epilepsy may undoubtedly be determined in a predisposed subject by a variety of peripheral stimuli. The writer has in one instance seen an attack precipitated by follicular tonsilitis on several successive occasions, and a recrudescence of quiescent epilepsy follow an attack of gastro-enteritis. All things considered, the treatment has considerable claims to be called rational. It is at least harmless, and on every ground well worthy of a trial.

[1] Intercolonial Med. Journ., Australasia, Nov. 1904.

